# System-Wide, Electronic Health Record–Based Medication Alerts for Appropriate Prescribing of Direct Oral Anticoagulants: Pilot Randomized Controlled Trial

**DOI:** 10.2196/64674

**Published:** 2024-11-08

**Authors:** Shawna N Smith, Michael S M Lanham, F Jacob Seagull, Morris Fabbri, Michael P Dorsch, Kathleen Jennings, Geoffrey Barnes

**Affiliations:** 1 Department of Health Management and Policy School of Public Health University of Michigan Ann Arbor, MI United States; 2 Michigan Program on Value Enhancement Institute for Healthcare Policy and Innovation University of Michigan Ann Arbor, MI United States; 3 Department of Learning Health Sciences University of Michigan Ann Arbor, MI United States; 4 Center for Bioethics and Social Sciences in Medicine University of Michigan Ann Arbor, MI United States; 5 College of Pharmacy University of Michigan Ann Arbor, MI United States; 6 Institute for Health Policy and Innovation University of Michigan Ann Arbor, MI United States; 7 Michigan Institute for Clinical and Health Research University of Michigan Ann Arbor, MI United States; 8 Division of Cardiovascular Medicine Department of Internal Medicine University of Michigan Ann Arbor, MI United States

**Keywords:** direct oral anticoagulants, electronic health record, medication safety, prescribing errors, pilot randomized controlled trial, alert system optimization, clinical decision support, EHR, randomized controlled trial, RCT, oral anticoagulants

## Abstract

**Background:**

While direct oral anticoagulants (DOACs) have improved oral anticoagulation management, inappropriate prescribing remains prevalent and leads to adverse drug events. Antithrombotic stewardship programs seek to enhance DOAC prescribing but require scalable and sustainable strategies.

**Objective:**

We present a pilot, prescriber-level randomized controlled trial to assess the effectiveness of electronic health record (EHR)–based medication alerts in a large health system.

**Methods:**

The pilot assessed prescriber responses to alerts for initial DOAC prescription errors (apixaban and rivaroxaban). A user-centered, multistage design process informed alert development, emphasizing clear indication, appropriate dosing based on renal function, and drug-drug interactions. Alerts appeared whenever a DOAC was being prescribed in a way that did not follow package label instructions. Clinician responses measured acceptability, accuracy, feasibility, and utilization of the alerts.

**Results:**

The study ran from August 1, 2022, through April 30, 2023. Only 1 prescriber requested trial exclusion, demonstrating acceptability. The error rate for false alerts due to incomplete data was 6.6% (16/243). Two scenarios with alert design and/or execution errors occurred but were quickly identified and resolved, underlining the importance of a responsive quality assurance process in EHR-based interventions. Trial feasibility issues related to alert-data capture were identified and resolved. Trial feasibility was also assessed with balanced randomization of prescribers and the inclusion of various alerts across both medications. Assessing utilization, 34.2% (83/243) of the encounters (with 134 prescribers) led to a prescription change.

**Conclusions:**

The pilot implementation study demonstrated the acceptability, accuracy, feasibility, and estimates of the utilization of EHR-based medication alerts for DOAC prescriptions and successfully established just-in-time randomization of prescribing clinicians. This pilot study sets the stage for large-scale, randomized implementation evaluations of EHR-based alerts to improve medication safety.

**Trial Registration:**

ClinicalTrials.gov NCT05351749; https://clinicaltrials.gov/study/NCT05351749

## Introduction

Oral anticoagulation is critical for the treatment of life-threatening conditions, including atrial fibrillation and venous thromboembolism. Since 2010, direct oral anticoagulants (DOACs) with more predictable anticoagulation effects have been broadly used for these indications. As such, DOACs are now recommended by guidelines as first-line therapy [1,2]. While easier to manage than the previously available anticoagulant (warfarin), they have some complex dosing rules that lead to inappropriate prescribing in up to 20% of patients [3]. In fact, anticoagulant medications are now the leading cause of adverse drug events in emergency departments in the United States [4,5]. To address this growing clinical challenge, antithrombotic stewardship programs have been proposed to reduce adverse drug events by improving appropriate, evidence-based prescribing of DOACs [6,7].

The traditional approach to anticoagulation care (developed for vitamin K antagonists) includes individual review of every patient’s clinical chart on a regular basis (eg, monthly). This model is unsustainable when individual health systems have more than 10,000-15,000 ambulatory patients using DOACs. As such, population health tools have been implemented, typically via the electronic health record (EHR), to improve the efficiency of clinical anticoagulation pharmacists reviewing charts and making prescribing changes [8,9]. However, several key questions remain regarding optimizing these review systems. One critical unanswered question addresses when a pharmacist is needed to manually review a patient’s chart versus when an automated message to a prescribing clinician could fix an inappropriate DOAC prescription. This is relevant both at the time an inappropriate prescription is ordered by the prescribing clinicians and over the long term, when other clinical factors change (eg, patient’s renal function declines 3 months after DOAC therapy was initiated, making it no longer appropriate).

Optimization studies have investigated the effects of individual intervention components (eg, DOAC pharmacist review) on the overall efficacy of the intervention package [10,11]. These kinds of studies can also help us to understand moderators of component effectiveness or under which circumstances or in which contexts resource-intensive components improve outcomes most, which is crucial to understand in developing scalable, sustainable interventions. To prepare for a larger trial [12] assessing the feasibility of implementing an EHR-based population health system and understand which clinical situations are most amenable to automated messages versus individual pharmacist review, we initiated a pilot, prescriber-level randomized controlled trial of DOAC medication alerts in a large health system. This trial includes just-in-time randomization of prescribers at the moment they place a DOAC order that triggers a prescribing alert, thus prospectively enrolling only prescribers who trigger alerts. In this paper, we report the findings from our pilot study. Importantly, we highlight the many lessons learned during this pilot phase that will impact how the future full-scale clinical trial is implemented and that are important for all scholars conducting EHR-based randomized controlled trials.

## Methods

### Overview

Our pilot study was designed to assess the feasibility and acceptability of 2 different EHR-based medication alerts sent directly to DOAC prescribers within 1 large health care system. While the full trial will test both medication alerts for initial DOAC prescribing errors and notifications for long-term prescribing errors [12], the pilot only focused on assessing responses to the initial alerts targeting new DOAC prescriptions. For the pilot, we further limited our assessment to 2 DOACs (apixaban and rivaroxaban), as they represent >95% of all DOACs prescribed in our health system and the United States.

### Alert Design

The medication alerts were designed using a multistage, user-centered design process using multiple contrasting prototypes. Several different prescribing clinicians of various career stages and specialties were involved in this process. The overarching principle of the design process was the 5 rights of health information technology: right information, right person, right format, right channel, and right time in the workflow [13].

Our user-centered design process also identified several unique aspects of DOAC medication alerts. First, there is a need to establish a clear indication for DOAC prescribing, as dosing differs by indication. Second, it is essential to clarify the dosing by renal function and sex assigned at birth, which uses the calculated Cockcroft-Gault creatine clearance estimation [14] according to the Federal Drug Administration (FDA) prescribing instructions. Third, the importance of drug-drug interactions is not well known by most clinicians. As such, the language explaining how drug-drug interactions may lead to an increase or decrease in DOAC drug level requires careful consideration [15].

### Pilot Study Design

This pilot study included all prescribers of DOAC medications in the ambulatory setting at Michigan Medicine from August 1, 2022, to April 1, 2023. Patients who received any inpatient or emergency department prescriptions of DOACs were excluded. Prescribers were included if the DOAC being prescribed was for stroke prevention in atrial fibrillation as identified through the act of associating a diagnosis with the medication order. Prescribers were enrolled into the study the first time that they attempted to sign a DOAC prescription that was inappropriate after the start of the study (types of inappropriate prescriptions are listed in Multimedia Appendix 1).

**Figure 1:**
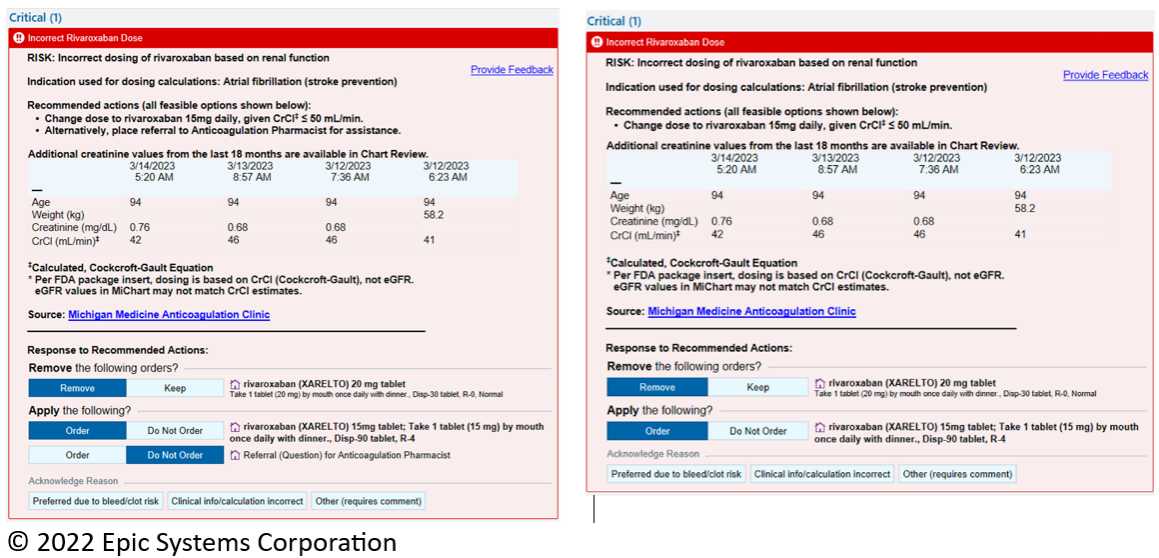
Examples of alerts in the pilot random controlled trial of electronic health record decision support. This alert indicates a potential problem in the dosing of the drug rivaroxaban to treat atrial fibrillation due to reduced renal function and is shown in the 2 experimental conditions, with a recommendation to consult a pharmacist (left) and without the recommendation to consult a pharmacist (right). CrC: creatinine clearance; eGFR: estimated glomerular filtration rate; FDA: Food and Drug Administration.

Once deemed eligible, prescribing clinicians were randomized (stratified by trainee or nontrainee and primary care or specialist status) with equal odds in real time to receive 1 of 2 different alert designs. All alerts included the same clinical information, but 1 alert also included an option to refer the prescription to the anticoagulation clinic for pharmacist assistance (Figure 1).

### Ethical Considerations

The University of Michigan Institutional Review Board approved this study (HUM00207165) and allowed a waiver of written documentation of consent, as well as allowing the informed consent process to be conducted through a priori notification. A notice about the trial was sent to all prescribing clinicians through a weekly EHR update email, explaining the possibility of receiving alerts or notifications. Prescribers could opt out in response to the message or at any point thereafter. This method of informed consent was necessary given that all prescribers of DOAC medications were potentially eligible participants but would not be enrolled in the study until they triggered an alert. This consent process posed minimal risk and was the least burdensome method for informed consent because the initiative was focused on improving the standard of care and did not involve the alerts triggering any action other than pointing out a potential prescription problem and suggesting alternatives. No compensation was provided to participants. No individual person’s data are included in this publication.

Additionally, our study empaneled a Data Safety and Monitoring Board, which reviewed any adverse events that occurred in patients within 30 days of an alert being triggered. The board provided safety oversight throughout the pilot study.

### Evaluation

We selected several key metrics to evaluate the pilot study. To assess the feasibility of provider and patient recruitment and randomization, we measured the number of DOAC medication alerts triggered per month and the number of clinicians who were appropriately randomized. To assess alert appropriateness, acceptability, and utilization, we measured the types of alerts triggered (ie, for which drug and which prescribing error[s]) and the different clinician responses to the alerts, categorized as leading to a behavior change or not leading to a behavior change. Within the prescription category, responses were grouped based on those that (1) selected the new medication order within the index alert (best practice alert); (2) selected the new medication order within the best practice alert after initially canceling and re-entering the order (sometimes multiple times); (3) canceled the initial order and placed a new order; and (4) canceled the initial order without any subsequent anticoagulant order. Following our full study protocol [12], which uses a 7-day window to assess any prescription change, we also assessed any situation in which an original order was canceled after receiving the alert to see if a new and “correct” order was placed within that window.

We also assessed the feasibility of our trial design by assessing the data collected in the EHR around clinician encounters with alerts. Lastly, we collected any clinician feedback about the alerts via the pharmacy team or standard EHR feedback channels. We also assessed the accuracy and appropriateness of the alerts for clinical encounters during which they were delivered. Finally, we audited EHR-captured data to assess whether all necessary metrics relevant to our outcomes were collected with fidelity.

## Results

**Figure 2:**
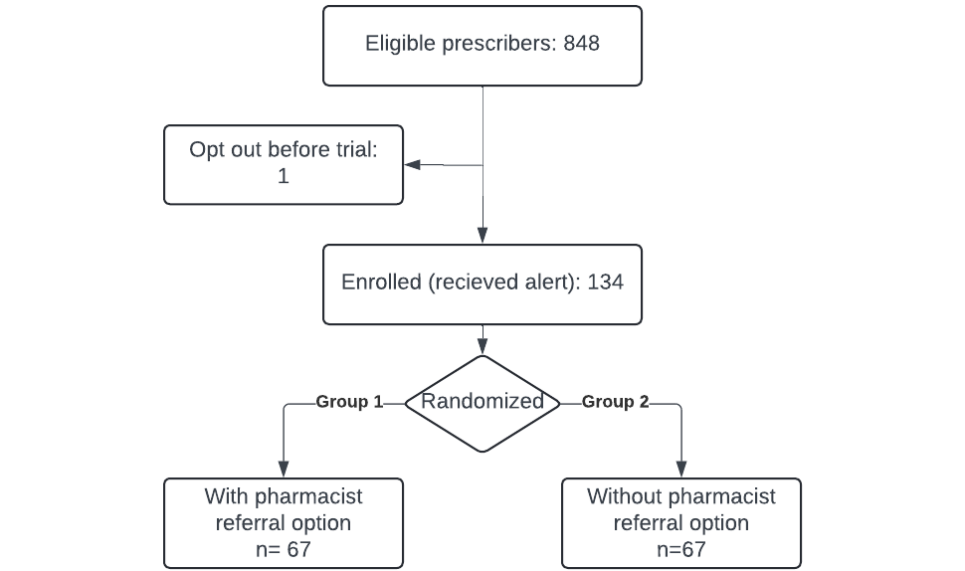
CONSORT (Consolidated Standards of Reporting Trials) diagram for pilot randomized controlled trial of electronic health record decision support for direct oral anticoagulant prescribing.

The pilot study ran from August 1, 2022, to April 30, 2023. During that time, we recorded 243 encounters with 134 providers (Table 1). There was a mean of 39.2 (SD 11.2) encounters per month and a mean of 1.8 (SD 1.4) encounters per prescriber during the study period. Only 1 clinician requested to be excluded from the trial; this occurred before the trial was initiated (Figure 2).

The most common alerts were for inappropriately low doses of apixaban (140/243, 57.6%; Table 2).

Alerts resulted in prescribing clinicians accepting the recommendation and changing the prescription in 34.2% (83/243) of encounters (Table 3).

**Table 1:** Composition of study population in the pilot randomized controlled trial of electronic health record clinical decision support of apixaban and rivaroxaban for atrial fibrillation, listing participants (number and percent) in the 2 experimental arms (with and without pharmacist referral) by clinical role.

Clinical role		Unique clinician participants (n=134), n (%)	With pharmacist referral option (n=67), n (%)	Without pharmacist referral option (n=67), n (%)
**Attending physician**		93 (69.9)	48 (71.6)	45 (67.1)
	Cardiology or other specialist	27 (29)	15 (31.3)	11 (24.4)
	Primary care	66 (71)	33 (68.7)	34 (75.6)
**Resident**		11 (8.3)	5 (7.5)	6 (9)
**Physician assistant**		6 (4.5)	6 (6)	2 (3)
**Nurse practitioner**		24 (18)	10 (14.9)	14 (20.9)

**Table 2:** The type of alerts observed in the pilot randomized controlled trial of electronic health record clinical decision support for apixaban and rivaroxaban for atrial fibrillation.

Alert encounter reason	Encounters (n=243), n (%)
Apixaban dose too low	140 (57.6)
Rivaroxaban dose too low	32 (13.2)
Apixaban dose too high	31 (12.8)
Rivaroxaban dose too high	31 (12.8)
Drug interaction	9 (3.7)
Total encounters	243 (100)

**Table 3:** Responses by prescribers to alerts for apixaban and rivaroxaban dosing issues when prescribed for atrial fibrillation in the pilot randomized controlled trial of electronic health record decision support.

Action			Response (n=243), n (%)
**Prescription changed**			83 (34.2)
	Changed order within BPA^a^ on initial alert	45 (18.5)	
	Changed order within BPA after multiple attempts	8 (3.3)	
	Canceled order, placed a new order outside BPA within 7 days	24 (9.9)	
	Canceled order, no new order placed within 7 days	6 (2.5)	
**Prescription unchanged**			160 (65.8)
	Overrode alert on initial alert	98 (40.3)	
	Overrode alert after multiple attempts	62 (25.5)	

^a^BPA: best practice alert.

Of the 6 cases where the initial order was canceled and no new order was placed within 7 days, 4 did not have a new order placed, 1 had a new order placed outside the 7-day window, and 1 involved a patient was given clinically appropriate verbal instructions to hold their anticoagulant temporarily while taking nirmatrelvir/ritonavir.

Two potential errors in our alerts were identified during the pilot trial. First, 1 clinician notified us that they believed the renal function calculation provided for their patient in the alert was inaccurate and did not match the estimate elsewhere in the EHR. Upon review, however, it was determined that this was not an error but rather reflected the difference between the creatinine clearance (reported in the alert) and the estimated glomerular filtration rate (reported elsewhere in the EHR). Noting that this issue could occur in future alerts, and to minimize confusion, we opted to add clarifying language to this particular alert that highlights the difference between the creatinine clearance (used to dose apixaban and rivaroxaban) and estimated glomerular filtration rate. A second potential error was brought to our attention during the study: 1 instance of an alert recommended removing an interacting medication (itraconazole) but no corresponding action button was provided. In response, we added this action button to the appropriate alert.

Second, we discovered that in some cases, there were critical elements related to prescribing that were not captured by the EHR, thus leading to misfiring alerts. The most common occurrence of this was medication orders that were not captured in discrete data element fields but rather entered as a free-text string. In these instances, the alert logic could not analyze the text string and these data were not easily captured in our reporting tools, resulting in missed or misfired alerts. For example, some alerts would specify a tablet strength in discrete data elements but report a total dose as a text string (eg, “take a half-tablet twice daily” and “take two pills together”). In these cases, as the alert logic runs only on discrete elements, it would not be able to correctly discern if the prescription is appropriate. While there is promise that real-time, reliable, natural language processing might be able to address such challenges in the near future, it was not available for this pilot study and therefore all data were included in the analysis. We performed a manual chart review for “false alarm” alerts, which revealed 6.6% (16/243) of alerts without complete entry of discrete data elements from this trial, resulting in a false alert. This provides an ecologically valid estimate of the “type 1” (false alarm) error rate for these alerts, demonstrating some inherent limits to alerting systems.

With respect to data, we discovered an EHR data capture problem, notably that when a prescribing clinician changed an (incorrect) order due to the alert firing, the EHR did not store the initial order. To address this concern, we redesigned the data capture mechanism to capture the data input when a DOAC medication order is first placed (when it triggers the alert but before it is finalized) instead of only recording the completed order, including both discrete and nondiscrete (free text) data elements in each order.

## Discussion

### Principal Findings

In this pilot study, we successfully implemented a prescriber-level, just-in-time randomization scheme within the EHR and enrolled 134 prescribers across 243 clinical encounters. More than one-third (83/243, 34.2%) of alerts resulted in prescribing clinician changing the original order, which provided strong support for the feasibility of our alerts. These alerts serve as a fail-safe mechanism to prevent potential prescribing errors from occurring and may be implemented in addition to any additional decision support tools that might reduce the incidence of prescribing errors.

Clinical alerts are often prone to high false alarm rates and are frequently ignored or overridden [16]. We achieved a high response rate by ensuring the validity of our alerts through thoughtful design, user testing, and selecting alerts that reflected clear consensus prescribing recommendations. For this reason, we chose to not include alerts regarding hepatic function, as such alerts lack clear consensus measures to guide dosing, and automatic alerts on hepatic guidelines would be difficult to implement effectively.

As this pilot study was not powered or designed to compare the 2 different alerts, we did not report an efficacy outcome. During the 9-month pilot study period, 1 programming error was identified and fixed, and 1 user-facing issue that required further clarification of the alert language was found. Additionally, the study team discovered that in situations where the triggering order was not signed by the prescriber, data on the order were not being recorded. The team made changes to alert programming to capture details about orders before signing to support analysis.

This pilot study demonstrated important feasibility aspects for operationalizing a clinician recruitment and randomization process directly within the EHR, as opposed to the randomization of all possible providers before the start of the study. This novel approach is key for ensuring appropriate and balanced randomization for trials of “just-in-time” alerts or other EHR-based communications where clinician actions may trigger interventions. In our case, clinicians were only randomized (and thus study eligible or enrolled) when they attempted to prescribe a non–evidence-based DOAC dose. This approach also avoided ongoing maintenance concerns as new providers were onboarded into the EHR. To a lesser extent, this study also speaks to the feasibility and acceptability of doing opt-out consents for system-wide, EHR-based implementation or optimization studies that allow for study enrollment upon the occurrence of triggering events, as only 1 clinician opted out before the start of the study and the only other feedback (discussed above) was related to the content of the alerts. This approach is critical for studies where the list of eligible participants (clinicians) is larger than the number of expected enrollees due to the unpredictable nature of clinician-patient interactions and clinician behavior, and where timely provision of alerts is required.

In addition to demonstrating the feasibility of the randomization and recruitment process and the acceptability of the alerts themselves, there were several important lessons learned during the pilot study that will improve the execution of our fully powered trial. First, multiple rounds of user feedback on contrasting designs were critical in developing clear alert language and design. However, even after this extensive user-centered design process, we found that some confusion—and need for clarification—remained. Specifically, despite careful planning and extensive expert input on the renal function language during the design process, at least 1 clinician required further clarification about the difference in renal function equation estimates for 1 of their patients. Our study design and team members were able to make a needed modification to continue to add clarity on behalf of the clinical users.

Second, despite careful review of all alerts by multiple study team members, programming errors and omissions are possible, and perhaps even likely, particularly when the number of possible tailored alerts is large (eg, 18 for this pilot study; Multimedia Appendix 1). Having dedicated information technology resources to identify and correct these errors quickly is essential for ensuring provider trust in the alerts and for accurately testing their effectiveness. Best practices for EHR-based studies should have a preplanned quality assurance process in place at the beginning of the trial, to determine how best to address these errors when identified and to remediate any identified errors as quickly as possible.

Third, EHR-based alert studies should schedule regular audits of alert appropriateness and comprehensiveness. This is especially true in clinical areas where new guidelines may be released regularly or new medications may change recommendations. We plan for a regular audit of any change in prescribing guidelines every 6 months during the full trial; this is when appropriate, data-driven audits of effectiveness should also be incorporated. The ability to collect data on the end-user experience and accurately report on the response to the medication alerts is essential, and some data may not be routinely stored in the EHR even if those data are essential to an alert action. One of the most important findings from this pilot study was the deficiencies in EHR data collection for evaluating the effectiveness of our alerts. For example, as noted above, we discovered that when clinicians were alerted to a prescribing error and that an alert resulted in a change to their prescription, the EHR did not store the initially entered order. From the perspective of the EHR, of course, this process makes sense because when an alert stopped the completion of an order entry, there is no final medication order to be stored and transferred to a pharmacy. But for our purposes, it prevented us from both (1) documenting prescribing errors as observed and (2) ensuring the fidelity of the alerts as triggered. To address this gap in data collection, we worked with information technology staff to change the way medication orders are captured in the EHR, ensuring that the initial order and any subsequent orders were captured, even if the initial order was not processed due to a subsequent order made in response to an alert.

Our analysis of “false alerts” caused by prescribers inputting free text to place medication orders shows that, while alerts can be useful in improving safe prescribing, no alerting system is perfect. Our “type 1” error rate (alerts that do not indicate an actual problem) remained low. Adjustments to our data collection methods and improvement of the alert logic should improve these rates and will be studied in more depth in our full clinical trial. In the future, EHRs may choose to limit or restrict the use of free-text prescribing to reduce errors and improve the application of clinical decision support systems.

There have been other published studies of clinical decision support of anticoagulant medications. In one example, a clinical decision support system was associated with a slight reduction in ischemic stroke and mortality as compared to usual care for patients treated with warfarin in Spain [17]. In another small example, the use of computerized clinical decision support for hospitalized patients was associated with a high level of recommendation adoption by clinicians [18]. However, few have provided prospective, randomized controlled trial evidence in the ambulatory setting for DOACs.

This pilot study had several strengths, including testing a novel, system-wide, real-time delivery of medication alerts; robust assessment of several key metrics related to feasibility, acceptability, and effectiveness; and a deep dive into EHR data collection as it relates to outcome assessment and high-fidelity alert delivery. In addition to these strengths, several limitations are also important to consider. First, as a pilot study, the aim is not to accurately describe the prescribing clinician’s reaction to the medication alerts or the comparison between the randomized groups. Rather, the aim is to assess the feasibility of randomization and recruitment while identifying and addressing potential design issues. Furthermore, the total number of alerts is significantly smaller than that of the intended full trial, and several of the less common alerts did not occur during the pilot phase. As such, future errors may develop during the full trial that must be quickly addressed. Finally, the pilot study focused only on alerts related to atrial fibrillation prescribing, delivered immediately upon attempting to prescribe. The full trial will include alerts for both atrial fibrillation and venous thromboembolism, along with notifications that occur after an initial prescription has been written [12]. While those elements were not tested directly in the pilot study, the lessons learned from the immediate atrial fibrillation alerts will be applied to the other venous thromboembolism alerts and the notifications that occur after the initial prescribing.

### Conclusions

In summary, this pilot study successfully implemented clinician-level, just-in-time randomization for medication alerts for inappropriate prescriptions of DOAC medications. Clinicians changed their behavior frequently in response to the alerts, and very few errors were identified over a 9-month pilot trial period. The subsequent full clinical trial will explore the impact of these medication alerts and 2 different types of long-term medication notifications across a wider range of clinical situations.

## Appendix

Multimedia Appendix 1 Types of alert included in the pilot random controlled trial of electronic health record decision support for apixaban and rivaroxaban prescribed for atrial fibrillation, and the logic that was used to determine whether an alert should be triggered.

Multimedia Appendix 2 CONSORT (Consolidated Standards of Reporting Trials) checklist.

## Abbreviations

DOAC: direct oral anticoagulant
EHR: electronic health record
FDA: Food and Drug Administration

## Data Availability

Datasets used in this study is available from the corresponding author upon reasonable request (deidentified).
