# Supplementary material for: System-Wide, Electronic Health Record–Based Medication Alerts for Appropriate Prescribing of Direct Oral Anticoagulants: Pilot Randomized Controlled Trial
Source: JMIR Form Res. 2024 Nov 8;8:e64674. doi: 10.2196/64674 (PMC11584537; doi:10.2196/64674)
Supplement: Multimedia Appendix 1 [file formative_v8i1e64674_app1.pdf]

Appendix 1: Types of alert and alert logic

Alert type	Medication	Class of alert	Specifics of alert	Recommended action
Apixaban dose too high	Apixaban	Wrong dose	Dosing criteria for 5 mg not met	Prescribe 2.5 mg BID
Apixaban dose too low	Apixaban	Wrong dose	Dosing criteria for 5 mg met	Prescribe 5 mg BID
Contraindication to any DOAC	Rivaroxaban	Contraindication – renal function	CrCl <15 ml/min	Switch to warfarin
	Rivaroxaban	Contraindication – renal function	Dialysis	Switch to warfarin
	Rivaroxaban	Contraindication - DDI	Combined inhibitor	Switch to warfarin
	Apixaban	Contraindication - DDI	Dosing criteria score low and combined inhibitor	Switch to warfarin
	Apixaban	Contraindication - DDI	Combined inducer	Switch to warfarin
Rivaroxaban dose too high	Rivaroxaban	Wrong dose	CrCl below 50 ml/min	Prescribe rivaroxaban 15 mg
Rivaroxaban dose too low	Rivaroxaban	Wrong dose	CrCl above 50 ml/min	Prescribe rivaroxaban 20 mg
	Rivaroxaban	Wrong dose	Dialysis	Prescribe rivaroxaban 20 mg
Drug interaction (rivaroxaban)	Rivaroxaban	Contraindication - DDI	Dosing criteria score low and combined inhibitor	Switch to apixaban 2.5 mg or warfarin
	Rivaroxaban	Contraindication - DDI	Dosing criteria score OK, moderate inhibitor	Switch to apixaban 2.5 mg or warfarin
	Rivaroxaban	Contraindication - DDI	Riva interaction with verapamil or dronederone	Switch to apixaban 2.5 mg or warfarin
	Rivaroxaban	Contraindication – renal function & DDI	CrCl 50 - 80 ml/min, combined inhibitor	Switch to dabigatran 150 mg or edoxaban 60 mg or warfarin
	Rivaroxaban	Contraindication – renal function & DDI	CrCl 30 - 50 ml/min, combined inhibitor	Switch to Dabigatran 75 mg or edoxaban 30 mg or warfarin
	Rivaroxaban	Contraindication – renal function & DDI	CrCl below 30 ml/min, combined inhibitor	Switch to edoxaban 30 mg or warfarin
	Rivaroxaban	Contraindication – renal function & DDI	CrCl below 80 ml/min, dosing criteria score low, moderate inhibitor	Switch to apixaban 5 mg or warfarin
	Rivaroxaban	Contraindication – renal function & DDI	CrCl below 80 ml/min, verapamil or dronederone	Switch to apixaban 5 mg or warfarin

BID: twice daily; CrCl: Creatinine Clearance; DDI: drug-drug interaction
